# Functional and pharmacological characterization of two different ASIC1a/2a heteromers reveals their sensitivity to the spider toxin PcTx1

**DOI:** 10.1038/srep27647

**Published:** 2016-06-09

**Authors:** Niko Joeres, Katrin Augustinowski, Andreas Neuhof, Marc Assmann, Stefan Gründer

**Affiliations:** 1 Institute of Physiology, RWTH Aachen University, Pauwelsstrasse 30, D-52074 Aachen, Germany

## Abstract

Acid Sensing Ion Channels (ASICs) detect extracellular proton signals and are involved in synaptic transmission and pain sensation. ASIC subunits assemble into homo- and heteromeric channels composed of three subunits. Single molecule imaging revealed that heteromers composed of ASIC1a and ASIC2a, which are widely expressed in the central nervous system, have a flexible 2:1/1:2 stoichiometry. It was hitherto not possible, however, to functionally differentiate these two heteromers. To have a homogenous population of ASIC1a/2a heteromers with either 2:1 or 1:2 stoichiometry, we covalently linked subunits in the desired configuration and characterized their functional properties in *Xenopus* oocytes. We show that the two heteromers have slightly different proton affinity, with an additional ASIC1a subunit increasing apparent affinity. Moreover, we found that zinc, which potentiates ASIC2a-containing ASICs but not homomeric ASIC1a, potentiates both heteromers. Finally, we show that PcTx1, which binds at subunit-subunit interfaces of homomeric ASIC1a, inhibits both heteromers suggesting that ASIC2a can also contribute to a PcTx1 binding site. Using this functional fingerprint, we show that rat cortical neurons predominantly express the ASIC1a/2a heteromer with a 2:1 stoichiometry. Collectively, our results reveal the contribution of individual subunits to the functional properties of ASIC1a/2a heteromers.

Acid Sensing Ion Channels (ASICs) are H^+^-gated Na^+^ channels and members of the degenerin/epithelial Na^+^ channel (DEG/ENaC) gene family^1^. Four different genes code for at least six different ASIC subunits^2^,^3^, which assemble into homo- or heteromeric channels^4^,^5^,^6^,^7^,^8^. Physiological functions of ASICs include synaptic transmission^9^,^10^ and pain sensation^11^,^12^,^13^,^14^,^15^,^16^,^17^. At synapses in the amygdala they contribute to long-term potentiation and fear conditioning^9^,^18^, making them candidates for drug targets for panic attacks and depression. Under pathological conditions, ASICs contribute to neuronal degeneration in an animal model of stroke^19^ and axonal degeneration in a model of neuronal inflammation^20^. Thus, ASICs are also promising drug targets for these pathologies.

Main ASICs in neurons of the central nervous system (CNS) are ASIC1a homomers, ASIC1a/2a heteromers^21^,^22^,^23^,^24^ and ASIC1a/2b heteromers^21^,^25^. The crystal structure of chicken ASIC1 revealed that it assembles into a homotrimer^26^,^27^ and a trimeric composition has recently been confirmed for ASIC1 in live cells^28^. Although many heteromeric ion channels, like nicotinic channels at the neuromuscular junction or glutamate receptors, have a fixed stoichiometry, it has recently been found that ASIC1a/2a heteromers have a flexible stoichiometry consisting of either two ASIC1a and one ASIC2a subunits or one ASIC1a and two ASIC2a subunits^28^. Thus, in cells co-expressing ASIC1a and ASIC2a, the two heteromers will co-exist in varying ratios. It was not possible to functionally differentiate the two heteromers in a single cell^28^. Since ASIC1a/2a heteromers are widely expressed in the CNS^21^,^22^,^23^,^24^, understanding their functional and pharmacological properties is important, however. For example, the spider toxin psalmotoxin 1 (PcTx1), a gating modifier of homomeric ASIC1^29^ that inhibits ASIC1a by slightly increasing its proton affinity shifting channels into the desensitized state at rest^30^, binds at subunit-subunit interfaces of two ASIC1a subunits^31^,^32^,^33^, highlighting that knowing the subunit composition is crucial for intelligent drug design.

To functionally differentiate the two ASIC1a/2a heteromers, we fixed the stoichiometry to either 2:1 or 1:2 by covalently linking individual subunits in the desired configurations. We then characterized their properties in *Xenopus laevis* oocytes using two-electrode voltage clamp. We determined apparent affinity for activation by protons and for steady-state desensitization, revealing that heteromers with two ASIC1a subunits have a slightly higher apparent proton affinity than heteromers with only one. The cation zinc is known to potentiate ASICs containing ASIC2a but not homomeric ASIC1a. We show that zinc has different effects on the two different heteromers. Finally, we show that the supposedly ASIC1-specific toxin PcTx1 inhibits both ASIC1a/2a heteromers, suggesting that it binds also at interfaces containing an ASIC2a subunit. In summary, our results reveal the functional and pharmacological properties of the two ASIC1a/2a heteromers and the contribution of individual subunits to these properties. Using this information, we show that rat cortical neurons mainly express the ASIC1a/2a heteromer with a 2:1 stoichiometry.

## Results

### The two ASIC1a/2a heteromers have slightly different apparent affinity for protons

**Figure 1:**
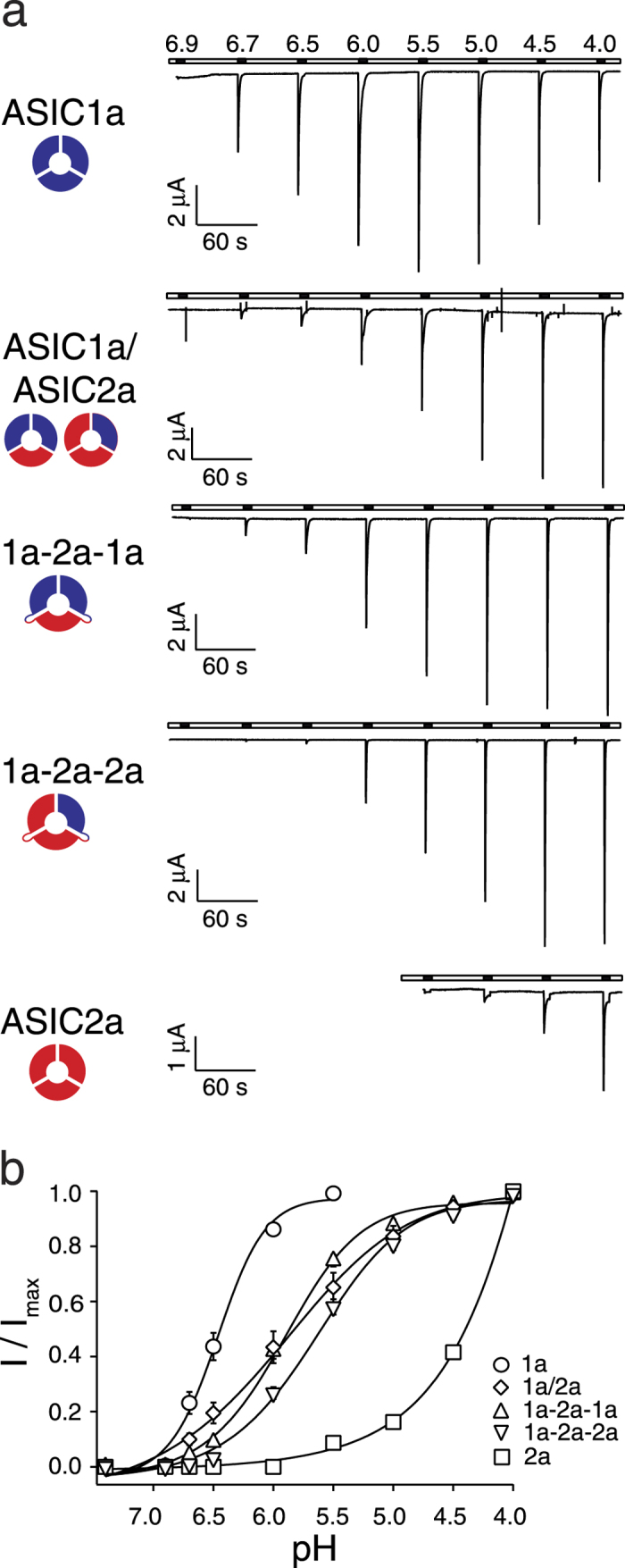
The two different ASIC1a/2a heteromers can be distinguished via their apparent affinity for protons. (**a**) Representative current traces of homo- and heteromeric ASICs, which were activated for 10 s with solutions of decreasing pH and allowed to recover for 50 s at pH 7.4. (**b**) Concentration-response curves of normalized currents. Maximal current amplitudes were 5.0 ± 0.8 μA (ASIC1a), 16.1 ± 2.7 μA (ASIC1a/2a), 13.3 ± 3.4 μA (ASIC1a-2a-1a), 11.3 ± 3.3 μA (ASIC1a-2a-2a), and 1.9 ± 0.3 μA (ASIC2a), respectively. The EC_50_ values of ASIC1a-2a-1a and ASIC1a-2a-2a were significantly different (p < 0.001). There was also a slight significant difference in the Hill-coefficients (p < 0.05). Data are reported as mean ± SEM (n = 12–17 individual oocytes).

To functionally characterize the two different ASIC1a/2a heteromers, we covalently linked ASIC1a and ASIC2a subunits in two different compositions: 1a-2a-1a and 1a-2a-2a, respectively (see Methods). First, we determined apparent affinities for protons of homomeric ASIC1a, homomeric ASIC2a and of ASICs in oocytes co-expressing ASIC1a and ASIC2a and compared them with the affinities of the two ASIC1a/2a concatamers (Fig. 1). The ASIC1a homomer had an apparent EC_50_ of pH 6.5 ± 0.04, a Hill-coefficient of 2.8 ± 0.2 (n = 17) and a time constant of desensitization τ_des_ (pH 4.0) of 1.7 ± 0.3 s (n = 16; Fig. 1B, Table 1). Homomeric ASIC2a was very different with an apparent EC_50_ of pH 3.8 ± 0.1, a Hill-coefficient of 1.0 ± 0.1 and τ_des_ (pH 4.0) of 1.6 ± 0.1 s (n = 13; Fig. 1B, Table 1). In addition, desensitization was incomplete after 10 s. Thus, apparent proton affinity of activation varied by almost three orders of magnitude between the two homomers. The Hill coefficient reflects the interaction between different subunits^34^, suggesting that the proton binding sites of ASIC1a have higher cooperativity than those of ASIC2a. Coinjection of cRNA of ASIC1a and ASIC2a at a ratio of 1:5 should lead to the predominant formation of heteromeric channels^28^. Activation curves of these ASICs could be fitted by a single Hill function with an apparent EC_50_ of pH 5.8 ± 0.1, a Hill-coefficient of 1.1 ± 0.1 and τ_des_ (pH 4.0) of 0.9 ± 0.1 s (n = 12; Fig. 1B, Table 1). The EC_50_ is between that of ASIC1a (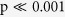) and ASIC2a (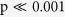). Together these values are in good agreement with earlier reports on these homo- and heteromeric ASICs^5^,^28^.

The ASIC1a-2a-1a concatamer had an apparent EC_50_ of pH 5.9 ± 0.04, a Hill-coefficient of 1.4 ± 0.1 and τ_des_ (pH 4.0) of 1.0 ± 0.2 s (n = 14; Fig. 1B, Table 1) while the ASIC1a-2a-2a concatamer had an apparent EC_50_ of pH 5.6 ± 0.04, a Hill-coefficient of 1.2 ± 0.1 and τ_des_ (pH 4.0) of 0.6 ± 0.1 s (n = 13; Fig. 1B, Table 1). Although the difference in apparent H^+^ affinity between the two concatamers was highly significant (p  < 0.001), it was rather slight (0.3 pH units), suggesting that the contribution of individual subunits to apparent H^+^ affinity is not simply additive but that apparent H^+^ affinity is rather determined by the complex interaction of all three subunits. The same reasoning applies to the slight difference in Hill-coefficients (p = 0.03). There was also only a slight difference in their time constants of desensitization τ_des_ (p = 0.06). Since apparent affinities of the two concatamers were so similar, it is not surprising that apparent affinity of each concatamer was not significantly different from that of ASICs of oocytes co-expressing ASIC1a and ASIC2a (p > 0.05) as these probably express a mix of both populations^28^. Similarly, time constants of desensitization τ_des_ of both concatamers were also not significantly different from those of oocytes co-expressing ASIC1a and ASIC2a (p > 0.05).

Importantly, while homomeric ASIC1a is robustly activated at pH 6.7, only the ASIC1a-2a-1a concatamer carried small currents at this pH. And while current amplitude of homomeric ASIC2a increased strongly at pH <4.5, activation of both concatamers was saturated at pH 4.5, (Fig. 1). These observations indicate that oocytes expressing the concatamers were not contaminated by homomeric ASICs.

**Figure 2:**
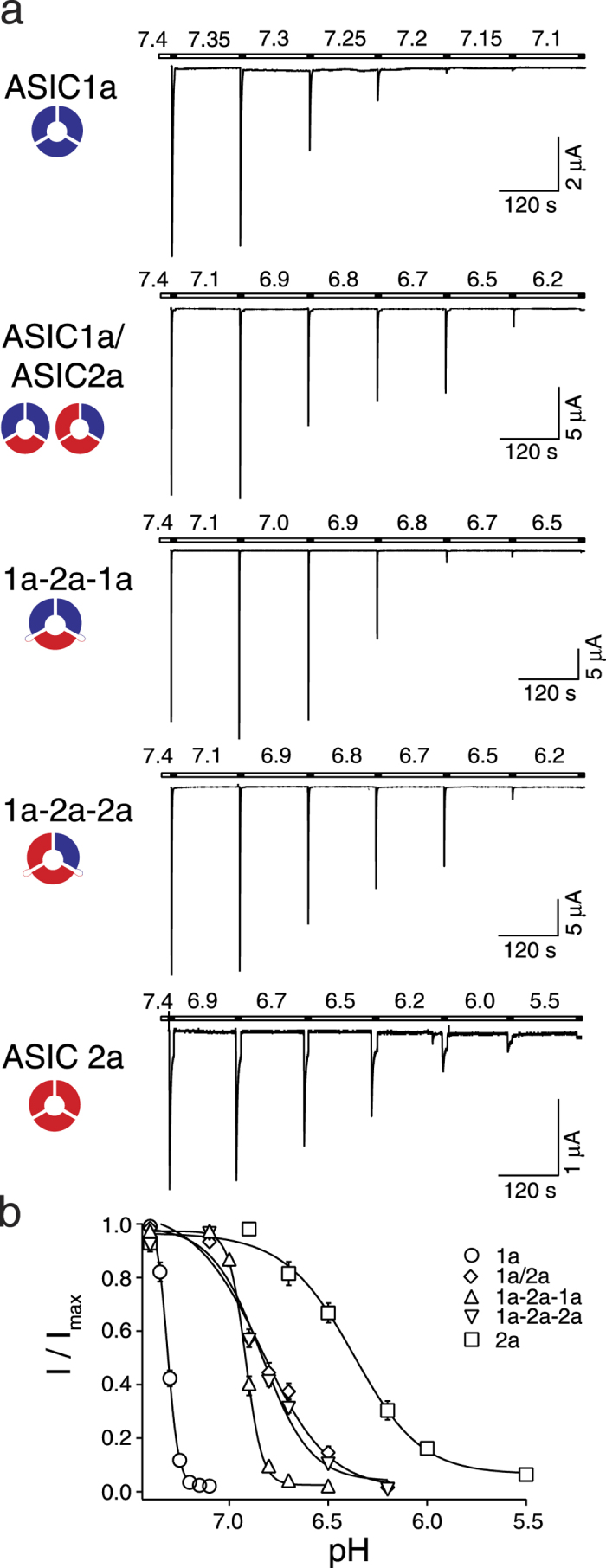
The two different ASIC1a/2a heteromers can be distinguished via their steady-state desensitization curves. (**a**) Representative current traces of homo- and heteromeric ASICs, which were activated for 10 s with pH 4.0 (pH 6.0 for ASIC1a) and allowed to recover for 120 s in a bath solution with decreasing pH. (**b**) Concentration-response curves of normalized currents. Maximal current amplitudes were 9.0 ± 2.2 μA (ASIC1a), 22.9 ± 2.3 μA (ASIC1a/2a), 25.7 ± 2.1 μA (ASIC1a-2a-1a), 12.5 ± 1.7 μA (ASIC1a-2a-2a), and 2.8 ± 0.4 μA (ASIC2a), respectively. The curves of both concatamers differ in their IC_50_ values (p < 0.001) as well as in their Hill-coefficients (p < 0.001), which were similar to that of the dominant ASIC form in the constructs. Data are reported as mean ± SEM (n = 8–16 individual oocytes).

We then determined steady-state desensitization curves. We activated oocytes with pH 4.0 for 10 s and let the channels recover in conditioning solutions of progressively increasing H^+^ concentrations until currents were no longer elicited (Fig. 2). ASIC1a was activated with pH 6.0 instead of pH 4.0 to avoid tachyphylaxis (see below)^35^. Homomeric ASIC1a had an apparent IC_50_ of pH 7.31 ± 0.005 with a Hill-coefficient of 13.9 ± 1.4 (n = 9), while ASIC2a had an apparent IC_50_ of pH 6.39 ± 0.03 with a Hill-coefficient of 2.6 ± 0.2 (n = 8). ASICs of oocytes co-expressing ASIC1a and ASIC2a had an apparent IC_50_ of pH 6.82 ± 0.03 with a Hill-coefficient of 2.9 ± 0.2 (n = 15; Fig. 2B, Table 1), in between the values of the two homomers (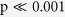).

The ASIC1a-2a-1a concatamer had an apparent IC_50_ of pH 6.91 ± 0.01 with a Hill-coefficient of 12.3 ± 1.6 (n = 15) and the ASIC1a-2a-2a concatamer had an apparent IC_50_ of pH 6.85 ± 0.02 with a Hill-coefficient of 3.7 ± 0.3 (n = 16; Fig. 2B, Table 1). Like for activation curves, the difference in apparent H^+^ affinity of steady-state desensitization, although highly significant (p < 0.001), was slight (0.06 pH units), further suggesting that the three subunits cooperate during H^+^ activation. Apparent IC_50_ of ASICs in oocytes co-expressing ASIC1a and ASIC2a was significantly different from that of the ASIC1a-2a-1a concatamer (p < 0.01) but not of the ASIC1a-2a-2a concatamer (p = 0.73). In contrast to apparent H^+^ affinity, the Hill-coefficient of the ASIC1a-2a-1a concatamer was similar to the Hill coefficient of homomeric ASIC1a (p = 0.51) and the Hill-coefficient of the ASIC1a-2a-2a concatamer was similar to the Hill coefficient of homomeric ASIC2a (p = 0.13). The difference between Hill-coefficients of the two concatamers was highly significant (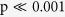).

Importantly, while homomeric ASIC1a is completely desensitized at pH 7.1, both concatamers where not desensitized at this pH (Fig. 2), and while homomeric ASIC2a is only partially desensitized at pH 6.5, both concatamers where almost completely desensitized at this pH (Fig. 2). These observations confirm that heteromers were the main configuration in oocytes expressing the concatamers.

### Tachyphylaxis is absent in all ASIC1a/2a heteromers

**Figure 3:**
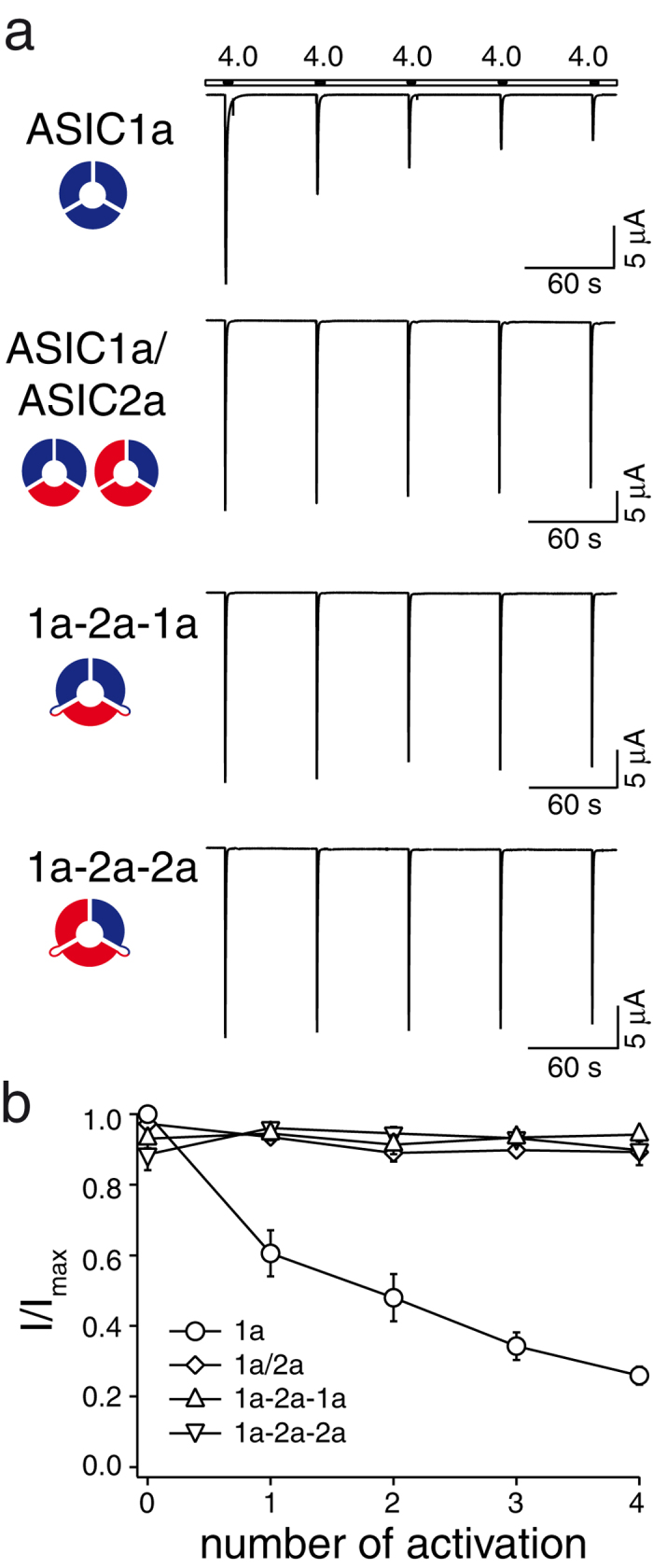
Tachyphylaxis is absent in ASIC1a/2a heteromers. ASICs were repeatedly activated for 10 s with pH 4.0 and allowed to recover for 50 s at pH 7.4. Only homomeric ASIC1a showed tachyphylaxis. Maximal current amplitudes were 30.6 ± 3.6 μA (ASIC1a), 26.2 ± 3.6 μA (ASIC1a/2a), 20.3 ± 5.0 μA (ASIC1a-2a-1a), and 15.1 ± 4.0 μA (ASIC1a-2a-2a), respectively. Data are reported as mean ± SEM (n = 4–9 individual oocytes; measurements for ASIC1a are from one week).

Repetitive activation of homomeric ASIC1a drives the channel in a long-lived inactive state, a process called tachyphylaxis^35^. In contrast, homomeric ASIC2a and ASICs of oocytes co-expressing ASIC1a and ASIC2a show no tachyphylaxis^35^. We explored tachyphylaxis of the two concatamers. As tachyphylaxis of ASIC1a is stronger at acidic pH^35^, we activated the channels repeatedly with pH 4.0 for 10 s and allowed them to recover for 50 s at pH 7.4. While homomeric ASIC1a showed strong tachyphylaxis under these conditions (Fig. 3), neither ASICs of oocytes co-expressing ASIC1a and ASIC2a nor any of the two concatamers showed tachyphylaxis (p > 0.05, ANOVA; Fig. 3), indicating that a single ASIC2a subunit in a heteromeric complex is sufficient to completely prevent tachyphylaxis. The absence of tachyphylaxis with both concatamers again excludes the presence of homomeric ASIC1a in oocytes expressing them.

### Currents through both ASIC1a/2a heteromers are potentiated by zinc

**Figure 4:**
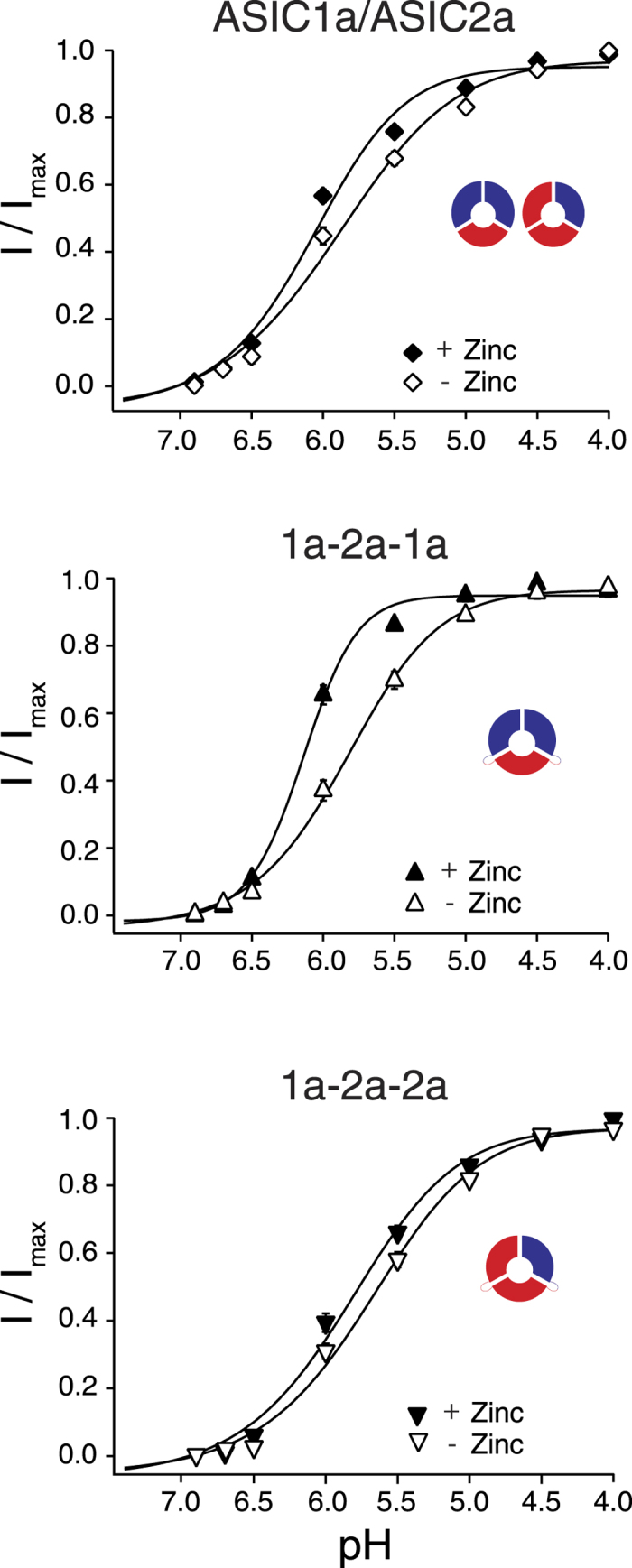
Both concatamers of ASIC1a/2a are potentiated by Zn^2+^. Concentration-response curves for normalized currents with and without Zn^2+^. The EC_50_ values of ASIC1a/2a (p = 0.045), ASIC1a-2a-1a (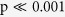) and ASIC1a-2a-2a (p < 0.01) were significantly left-shifted in the presence of 300 μm Zn^2+^. There was also a significant increase in the Hill-coefficient of ASIC1a-2a-1a (p < 0.001) and ASIC1a/2a (p < 0.01) but not of ASIC1a-2a-2a (p = 0.8). Maximal current amplitudes were 14.5 ± 3.1 μA (ASIC1a/2a), 20.3 ± 3.5 μA (ASIC1a/2a + Zn^2+^), 14.2 ± 3.1 μA (ASIC1a-2a-1a), 17.6 ± 3.4 μA (ASIC1a-2a-1a + Zn^2+^), 12.3 ± 1.8 μA (ASIC1a-2a-2a) and 11.9 ± 3.3 μA (ASIC1a-2a-2a + Zn^2+^), respectively. Data are reported as mean ± SEM (n = 10–13 individual oocytes).

At micromolar concentrations, the metal ion zinc (Zn^2+^) potentiates currents of ASIC2a homomers and ASIC1a/2a heteromers but not of ASIC1a homomers^36^. For ASIC1a/2a heteromers, it shifts activation curves to the left and increases the Hill-coefficient^36^. We explored whether the two concatamers are differentially modulated by Zn^2+^. One group of oocytes was activated by H^+^ without Zn^2+^ and one group with a co-application of H^+^ and 300 μmol Zn^2+^. Like previously reported^36^, for oocytes co-expressing ASIC1a and ASIC2, Zn^2+^ significantly shifted activation curves (EC_50_, from pH 5.9 ± 0.1 without Zn^2+^ to pH 6.1 ± 0.03 with Zn^2+^; p = 0.04; Fig. 4, Table 2) and increased the Hill-coefficient from 1.0 ± 0.1 to 1.4 ± 0.1 (p < 0.01; n = 10–12; Table 2). Similarly, for the ASIC1a-2a-1a concatamer, Zn^2+^ significantly shifted the EC_50_ from pH 5.8 ± 0.04 to 6.1 ± 0.02 (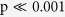; Fig. 4, Table 2) and increased the Hill-coefficient from 1.4 ± 0.1 to 2.2 ± 0.1 (p < 0.001; n = 12). For the ASIC1a-2a-2a concatamer, Zn^2+^ also significantly shifted the EC_50_ from pH 5.7 ± 0.04 to 5.9 ± 0.03 (p < 0.01; Fig. 4, Table 2) but did not increase the Hill-coefficient (p = 0.8; n = 12; Table 2). These data show that one ASIC2a subunit in a complex is sufficient for potentiation by Zn^2+^ but surprisingly it seems that two ASIC1a subunits are necessary for an increase of the Hill coefficient by Zn^2+^.

### Psalmotoxin 1 inhibits heteromeric ASIC1a/2a

The tarantula toxin psalmotoxin 1 (PcTx1) inhibits ASIC1a^30^,^37^ and stabilizes the open state of ASIC1b^29^. It is currently believed that PcTx1 specifically inhibits homomeric ASIC1^37^; only for ASIC1a/2b heteromers has it been shown that they are also inhibited by PcTx1^25^. Co-crystals of cASIC1 with PcTx1 have shown that PcTx1 binds to the extracellular domain at subunit interfaces of ASIC1^31^,^32^,^33^, suggesting that the 1a-2a-1a concatamer, which has one ASIC1a-ASIC1a interface, also binds PcTx1 while the 1a-2a-2a concatamer, which lacks such an interface, might not bind PcTx1.

**Figure 5:**
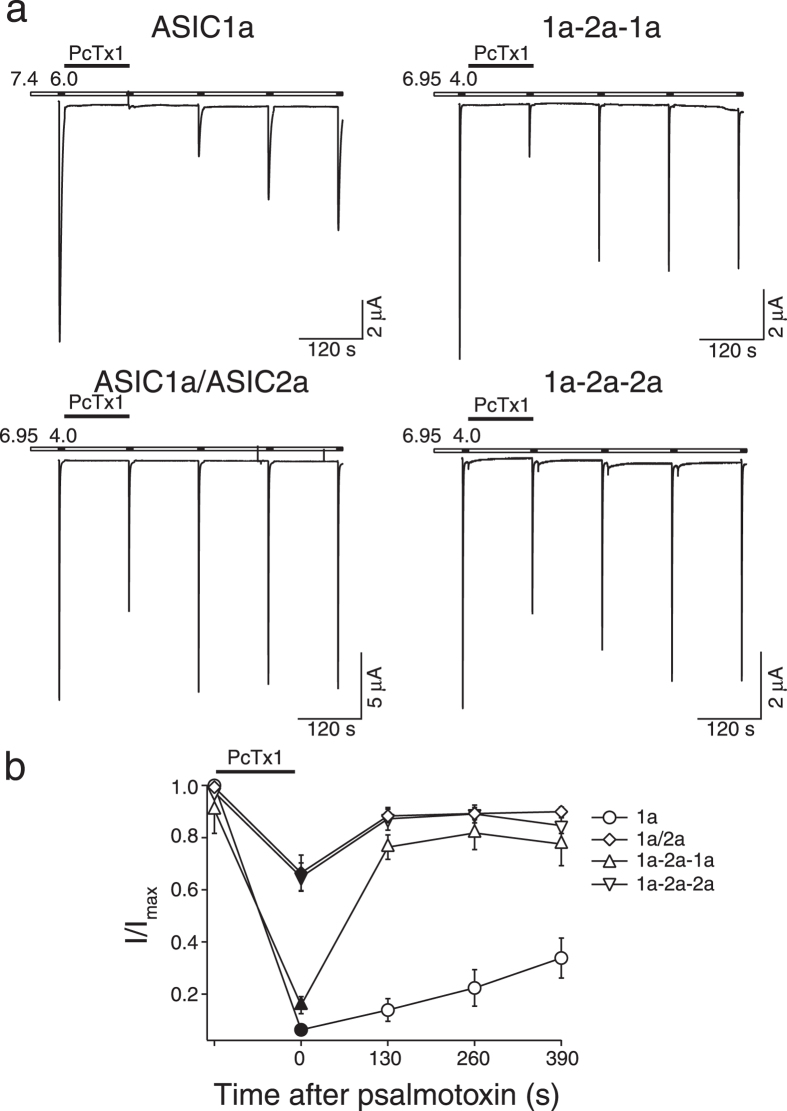
Inhibition by PcTx1 can distinguish between the two ASIC1a/2a heteromers. (**a**) Representative current traces showing the inhibition of different ASICs by PcTx1. ASICs were activated with pH 4.0 (pH 6.0 for homomeric ASIC1a) for 10 s and allowed to recover for 120 s in pH 6.95 (pH 7.4 for ASIC1a). 50 nM PcTx1 was applied in the conditioning solution before the second activation. (**b**) Comparison of the inhibition by PcTx1 of the different ASICs. The difference between ASIC1a-2a-1a and ASIC1a-2a-2a (p < 0.001) as well as the difference between ASIC1a-2a-1a and ASIC1a/2a (p < 0.001) were significant. There was no significant difference between ASIC1a/2a and ASIC1a-2a-2a. Maximal current amplitudes were 9.6 ± 1.9 μA (ASIC1a), 15.2 ± 1.7 μA (ASIC1a/2a), 12.9 ± 2.8 μA (ASIC1a-2a-1a), and 9.1 ± 1.4 μA (ASIC1a-2a-2a), respectively. Data are reported as mean ± SEM (n = 8–10).

Usually inhibition by PcTx1 is explored at a conditioning pH of 7.4, but as PcTx1 inhibits ASIC1 by shifting steady-state desensitization curves, inhibition by PcTx1 is strictly pH-dependent^30^. We therefore tested inhibition by PcTx1 of heteromeric ASICs at a conditioning pH of 6.95, a pH value at which desensitization just starts (Fig. 2); for ASIC1a we used conditioning pH 7.4. We activated heteromers for 10 s with pH 4.0 (ASIC1a with pH 6.0) and let them recover at pH 6.95 (7.4 for ASIC1a) in the presence of 50 nM PcTx1, followed by repetitive activations with pH 4.0 (6.0 for ASIC1a) in the absence of PcTx1 (Fig. 5). PcTx1 reduced ASIC1a currents to 6.3 ± 1.2% which incompletely recovered to 33.8 ± 7.6% of the first current amplitude after 390 s (n = 8); this incomplete recovery from inhibition is at least partially due to tachyphylaxis^35^. For oocytes expressing ASIC1a and ASIC2a, PcTx1 reduced the current to 66.5 ± 6.8% of the first amplitude (n = 10). Strikingly, the ASIC1a-2a-1a concatamer was similarly inhibited as homomeric ASIC1a: PcTx1 reduced currents to 15.7 ± 3.3% (n = 8; Fig. 5). Even more surprisingly, PcTx1 also inhibited currents of the ASIC1a-2a-2a concatamer, which has no ASIC1a-ASIC1a interface, to 64.8 ± 5.4% (n = 8; Fig. 5). Although the difference in inhibition by PcTx1 between the ASIC1a-2a-1a and the ASIC1a-2a-2a concatamer was statistically significant (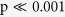), the inhibition of the ASIC1a-2a-2a concatamer by PcTx1 strongly suggests that PcTx1 also binds at interfaces between ASIC1a and ASIC2a subunits.

### Characterization of heteromeric ASIC1a/2a in rat cortical neurons

**Figure 6:**
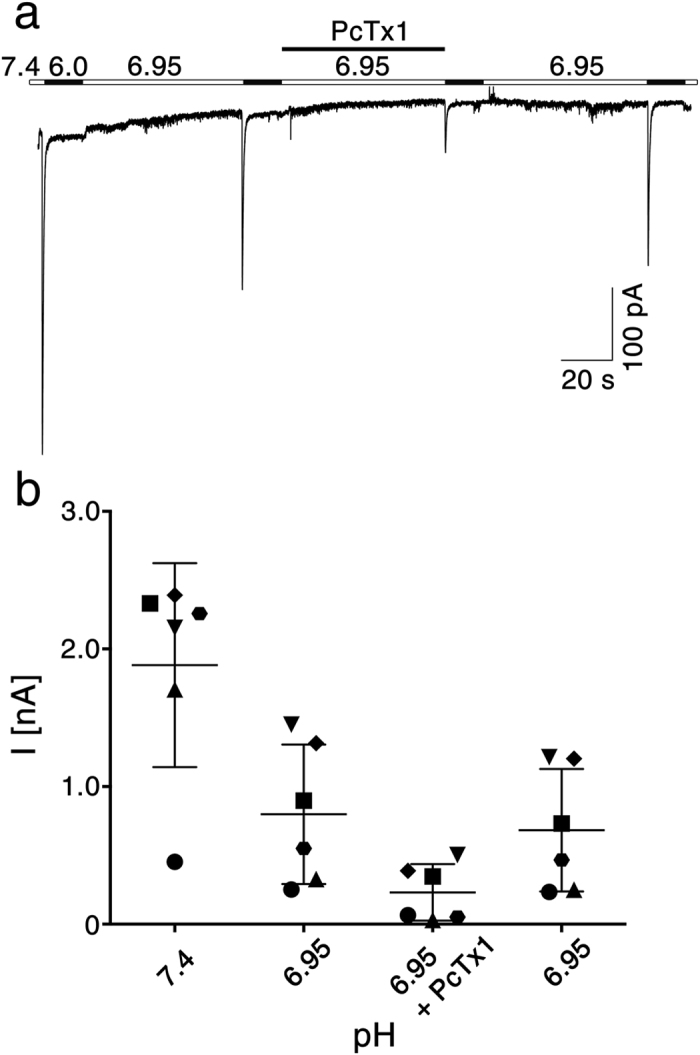
PcTx1 inhibits ASIC1a/2a heteromers in rat cortical neurons. (**a**) Representative current trace showing the inhibition of ASICs by PcTx1 at conditioning pH 6.95. ASICs were activated with pH 6.0 for 15 s and allowed to recover for 60 s in pH 6.95. 50 nM PcTx1 was applied in the conditioning solution before the third activation. (**b**) Quantitative analysis of six measurements like the one shown in (**a**). Symbols represent six individual neurons, bars represent mean and SD, respectively. Conditioning pH 6.95 desensitized homomeric ASIC1a and heteromeric ASIC1a/2b. Co-application of PcTx1 revealed the PcTx1-sensitivity of the remaining ASIC1a/2a heteromers in these neurons.

The most prominent functional difference between the two ASIC1a/2a heteromers was the differential inhibition by PcTx1 at conditioning pH 6.95. We asked whether we could use this information to identify the ASIC1a/2a heteromer expressed in cortical neurons. As we used rat ASICs for heterologous expression, we isolated cortical neurons from rats and characterized their ASICs. At conditioning pH 7.4 and a holding potential of −70 mV, pH 6.0 elicited robust transient inward currents with a mean amplitude of 1.88 ± 0.68 nA (n = 6; Fig. 6). Reducing conditioning pH to 6.95, which completely desensitizes homomeric ASIC1a (Fig. 2) and heteromeric ASIC1a/2b^25^,^38^, reduced current amplitude to 0.80 ± 0.46 nA (n =  6; Fig. 6). Considering that the two ASIC1a/2a heteromers are ~30% desensitized at conditioning pH 6.95 (Fig. 2) and that they are only ~50% activated by pH 6.0 (Fig. 1 and Table 1), this result suggests that >50% of the ASICs in rat cortical neurons were heteromeric ASIC1a/2a. Application of 50 nM PcTx1 for 60 sec at pH 6.95 further reduced current amplitude to 0.23 ± 0.19 nA (p < 0.01, n =  6; Fig. 6), which recovered within 60 sec to 85% (0.68 ± 0.40 nA; Fig. 6). This reduction of the ASIC current by PcTx1 at pH 6.95 to 25 ± 11% is close to the value determined for the 1a-2a-1a concatamer in oocytes (15.7 ± 3.3%), suggesting that the majority (~80%) of heteromeric ASIC1a/2a in these neurons had the 1a-2a-1a configuration. The inhibition by PcTx1 of heteromeric ASIC1a/2a in these cortical neurons varied from 61% to 91% (Fig. 6B), suggesting that the cells might contain varying amounts of the two heteromers, but the number of cells measured (n = 6) was too small to draw firm conclusions.

## Discussion

A recent study reported that ASIC1a/2a heteromers assemble in two different stoichiometries, but that the two heteromers cannot be functionally differentiated when present in the same cell^28^. In the present work we therefore used covalently linked ASIC1a and ASIC2a subunits to characterize the properties of the two ASIC1a/2a heteromers separately in different cells. Our results confirm that the two heteromers have surprisingly similar functional properties, rendering their functional differentiation in the same cell very difficult. In addition, they suggest that the properties of ASICs from oocytes co-expressing ASIC1a and ASIC2a indeed arise from a mixture of the two individual heteromers.

Comparison of functional properties - such as activation at pH 6.7 and pH 4, steady-state desensitization at pH 7.1 and 6.5, and tachyphylaxis - of ASICs from oocytes expressing the concatamers with those from oocytes expressing homomeric ASIC1a and ASIC2a largely excludes the substantial presence of homomeric assemblies in oocytes expressing the concatamers, strongly suggesting that these oocytes indeed expressed only the desired concatamers, allowing their faithful characterization.

As expected, apparent proton affinity for activation and steady-state desensitization were significantly higher for the ASIC1a-2a-1a concatamer than the ASIC1a-2a-2a concatamer. But as EC_50_ values of activation vary by almost three pH units between homomeric ASIC1a and ASIC2a (Fig. 1) the rather slight difference in apparent affinity of the concatamers (0.2 pH units) was surprising. Desensitization likely masks the real potency of protons at ASICs and steady-state desensitization therefore more faithfully reports potency of protons at ASICs^39^. IC_50_ values of steady-state desensitization vary by one pH unit between homomeric ASIC1a and ASIC2a (Fig. 2) and the comparably slight difference in IC_50_ of the concatamers (0.06 pH units) suggests that the contribution of individual subunits is indeed not simply additive. Similarly, the Hill coefficient varies strongly between ASIC1a and ASIC2a, from 13.9 to 2.8. The Hill coefficient is best described as an interaction coefficient, reflecting the extent of positive cooperativity among multiple binding sites^34^. Thus, the large difference in Hill coefficients suggests that H^+^ binding sites of ASIC1a have a much higher cooperativity than those of ASIC2a. The ASIC1a-2a-1a concatamer had a Hill coefficient of 12.3, not much different from homomeric ASIC1a, while the ASIC1a-2a-2a concatamer had a Hill coefficient of 3.65, not much different from homomeric ASIC2a, arguing that two ASIC1a subunits in a complex introduce high cooperativity of H^+^ binding sites. The large difference in Hill coefficients between the two concatamers is another indication that oocytes injected with the concatamers indeed expressed separate heteromers.

While a single ASIC1a subunit in the trimeric complex did not strongly increase the Hill coefficient, it comparatively strongly increased the apparent H^+^ affinity, suggesting that the apparent affinity for protons of an ASIC is not simply determined by cooperativity of H^+^ binding sites, but that the higher apparent proton affinity of ASIC1a arises, at least in part, from facilitated conformational changes triggered by protons. In this interpretation, the first ASIC1a subunit in a trimeric complex would rather strongly facilitate conformational changes associated with open gating while a second ASIC1a subunit had only a small further effect. A third ASIC1a subunit, in contrast, had again a large facilitating influence.

Tachyphylaxis reduces ASIC1a activity with repeated activation, especially at low pH^35^. It is due to a long-lived inactive state^35^,^40^. Our results confirm that tachyphylaxis is unique to homomeric ASIC1a. Permeating protons appear to be important for tachyphylaxis^35^ and the finding that three ASIC1a in a complex are necessary for tachyphylaxis might be useful to uncover its structural basis.

Zn^2+^ potentiates currents carried by homomeric ASIC2a and heteromeric ASICs containing ASIC2a^36^. While potentiation of homomeric ASIC2a is moderate, heteromeric ASIC1a/2a is more strongly potentiated by Zn^2+^ and potentiation of the heteromer is at least partially due to an increase in the Hill coefficient^36^. His-162 and His-339 in the extracellular domain of ASIC2a are important for potentation by Zn^2+^ 36, probably by contributing to a Zn^2+^ binding site. Our results show that Zn^2+^ shifts the dose-response curves of both ASIC1a-2a-1a and ASIC1a-2a-2a. However, Zn^2+^ increased only the Hill-coefficient of ASIC1a-2a-1a and not of ASIC1a-2a-2a. Thus, it appears that ASIC2a binds Zn^2+^ but that two ASIC1a subunits have to be present to allow Zn^2+^ to increase cooperativity of H^+^ binding sites, such that Zn^2+^ has a predominant effect on the ASIC1a-2a-1a heteromer.

Finally, we showed for the first time that PcTx1 inhibits also ASIC1a/2a heteromers. Usually, inhibition by PcTx1 is investigated at conditioning pH 7.4. At this pH, ASIC1a/2a heteromers will not be inhibited because their steady-state desensitization curves are shifted towards more acidic pH, explaining why inhibition by PcTx1 of ASIC1a/2a heteromers has not been reported previously. Homomeric ASIC1a and heteromeric ASIC1a/2b, in contrast, start to desensitize at or slightly below pH 7.4 25,30. Besides apparent IC_50_ of steady-state desensitization curves, the Hill coefficient of steady-state desensitization determines the extent of inhibition by PcTx1, because a larger Hill coefficient reflects a stronger dependence of steady-state-desensitization on pH. Thus, a shift by the same pH value will more strongly desensitize an ASIC when the Hill coefficient is large. It is therefore expected that the 1a-2a-1a concatamer (Hill coefficient = 12.3; Table 1) will be more strongly inhibited by PcTx1 than the 1a-2a-2a concatamer (Hill coefficient = 3.6; Table 1), as was indeed observed (Fig. 5). To estimate whether the different inhibition by PcTx1 of the two concatamers (Fig. 5) can be explained solely by their different Hill coefficients, we calculated (see Methods) from the fits to the measured steady-state desensitization curves (Fig. 2) the shift necessary to account for the observed current reduction by 50 nM PcTx1 (Fig. 5). This calculation yielded an expected shift of 0.16 ± 0.03 pH units for oocytes expressing a mix of the two heteromers, of 0.12 ± 0.03 pH units for the ASIC1a-2a-1a concatamer and of 0.16 ± 0.12 pH units for the ASIC1a-2a-2a concatamer. Thus, according to these calculations, PcTx1 would shift steady-state desensitization more strongly for the ASIC1a-2a-2a concatamer than the ASIC1a-2a-1a concatamer, despite a smaller inhibition. It, thus, appears that PcTx1 also binds at interfaces between one ASIC1a and one ASIC2a subunit and perhaps also between two ASIC2a subunits. Binding at these interfaces would increase apparent affinity for protons as it does for homomeric ASIC1a^30^.

**Figure 7:**
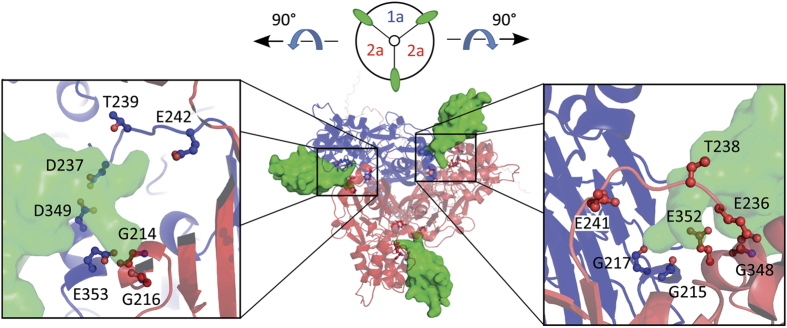
Possible interactions of PcTx1 with subunit interfaces of the ASIC1a-2a-2a heteromer. The homology model of the heteromer with ASIC1a (blue) and ASIC2a (red) is shown in cartoon and PcTx1 (green) in solvent-accessible surface representation. Boxes illustrate the amino acids (stick and spheres representation) in the ASIC1a/2a subunit interfaces that interact with PcTx1, namely Gly214, Gly216, Glu236, Thr238, Glu241, and Glu352 in rASIC2a and Gly215, Gly217, Asp237, Thr239, Glu242, Asp349, and Glu353 in rASIC1a. Five interacting amino acids are completely conserved between cASIC1, rASIC1a and rASIC2a. Numbers of amino acids refer to rASIC1a and rASIC2a, respectively.

Using the structure of cASIC1 in complex with PcTx1^31^, we generated a homology model of the ASIC1a-2a-2a heteromer (Fig. 7) to inspect the putative PcTx1 binding sites. Both interfaces between an ASIC1a and an ASIC2a subunit in the 1a-2a-2a heteromer indeed carry the amino acids, which are crucial for the interaction of cASIC1 with PcTx1^31^,^32^,^33^. Seven amino acids of cASIC1 interact closely with PcTx1 (Gly216, Gly218, Asp238, Thr240, Glu243, Asp350, and Glu354 in cASIC1), either by H-bonds or interactions with backbone oxygens^31^,^32^. Five out of these seven amino acids are conserved in ASIC2a (Gly214, Gly216, Thr238, Glu241, and Glu352 in rASIC2a). Additionally, Glu236 of ASIC2a, which is at a position corresponding to Asp238 of cASIC1, interacts with PcTx1 in our modelled heteromer (Fig. 7). Only Gly348 of ASIC2a, which replaces Asp350 of cASIC1, does not contribute to stabilize PcTx1 binding (Fig. 7), but does also not interfere with PcTx1 binding. The conservation of important interacting amino acids in ASIC2a is in agreement with the idea that PcTx1 also binds to ASIC1a-ASIC2a interfaces.

ASIC1a and ASIC2a assemble randomly into homo- and heteromeric channels^28^. Thus, increasing the expression level of ASIC2a relative to ASIC1a should gradually increase the population of heteromeric channels with a 1a-2a-1a configuration, followed by the population with a 1a-2a-2a configuration and finally also of homomeric ASIC2a. In a recent study, the relative expression of ASIC1a and ASIC2a in different regions of the mouse brain has been assessed and, assuming random assembly, the relative proportion of ASICs with different stoichiometries calculated^41^. This suggested that approximately 60% of the ASICs in cortical neurons are heteromeric ASIC1a/2a, and that 73% of these have a 1a-2a-1a configuration and the other 27% a 1a-2a-2a configuration^41^. These results are in good agreement with our results, which suggest that in rat cortical neurons >50% of the ASICs are heteromeric ASIC1a/2a and of these ~80% have the 1a-2a-1a configuration. Thus, our results are in agreement with the idea that ASIC1a/2a heteromer with a 2:1 stoichiometry is much more abundant than the heteromer with a 1:2 stoichiometry^41^. This situation may well vary, however, and our characterization of the two ASIC1a/2a heteromers will be useful to determine their abundance in individual neurons.

In summary, although the characteristics of the two ASIC1a/2a heteromers are surprisingly similar, we show that they can be separated functionally and pharmacologically, which can be used to differentiate them *in situ*. Moreover, our data reveals that individual subunits do not contribute equally to the properties of heteromeric ASICs, highlighting a complex interaction of subunits to gate an ASIC.

## Methods

### Molecular biology

We generated concatamers covalently joining three coding sequences of either rat ASIC1a or rat ASIC2a using recombinant PCR. ASIC sequences were joined via a linker containing the sequence Asn-Asn-Asn-Asp-Ile-Asn-Asn with five asparagine residues at their margins and an EcoRV restriction site in their centres.

### Electrophysiology

Electrophysiological measurements were conducted as previously described^30^,^42^ by expression of ASICs in stage V and VI oocytes of *Xenopus laevis*. Oocytes were surgically removed under anaesthesia (4 g l^−1^ tricaine methanesulfonate for 20–30 min) from adult *Xenopus laevis* females. Animal care and experiments were conducted under protocols approved by the State office for nature, environment and consumer protection (LANUV) of the State North Rhine-Westphalia (NRW) and were performed in accordance with LANUV NRW guidelines. 0.03 to 3 ng of *in vitro* synthesized cRNA (SP6 mMessage mMaschine kit, Ambion, Austin Texas, USA) was injected in oocytes which were incubated for 1 to 2 days at 19 °C in OR-2 medium which contains (in mM): 82.5 NaCl, 2.5 KCl, 1.0 Na_2_HPO_4_, 1.0 MgCl_2_, 1.0 CaCl_2_, 5.0 HEPES, 0.5 g/l PVP, 1,000 U/l penicillin and 10 mg/l streptomycin, pH 7.3. For expression of heteromeric ASIC1a/2a, cRNAs of rat ASIC1a and rat ASIC2a were injected at a ratio of 1 to 5 28. Currents were measured using two-electrode voltage clamp (TEVC) with the oocyte testing carousal (OTC) and a pump driven solution exchange system (npi electronic GmbH, Tamm, Germany). Solution exchange and carousal movement were controlled using the software Cellworks 5.1.1 (npi electronic). Data was acquired with a TurboTec 03X amplifier (npi electronic) and sampled at 0.1 to 1 kHz and filtered at 20 Hz. Bath solution contained (in mM): 140 NaCl, 1.8 CaCl_2_, 1.0 MgCl_2_, 10 HEPES; holding potential was −70mV. pH was adjusted using NaOH; MES replaced HEPES buffer at pH <6.7. Psalmotoxin 1 was from Alomone Labs (Jerusalem, Israel). If not specified otherwise, we measured at least 3 oocytes per week and repeated all experiments in two weeks. All measurements of ASIC1a/2a and the concatamers were done in the same weeks.

Native cortical neurons were isolated from 1-day old Wistar rats (Charles River). Rats were decapitated and whole brains rapidly removed and stored on ice-cold Hanks´ balanced salt solution (HBSS; Pan Biotech). Small pieces of cortices were dissected, transferred into a 15 ml tube and incubated for 9 min at 37 °C in 2 ml HBSS containing 0.015% trypsin from bovine pancreas (Sigma). Cells were then mechanically dissociated using two fire-polished glass pipettes and plated on cover slips coated with poly-l-ornithine and laminin in 35-mm dishes. Neurons were cultured with neuro-basal medium supplemented with B27 and maintained at 37 °C and 5% CO_2_. Cultures were fed twice a week and used for patch-clamp recordings after 12 days in culture. Animal care and experiments followed approved institutional guidelines at RWTH Aachen University. The bath solution for the cortical neurons contained (in mM): 100 NaCl, 5.4 KCl, 10 HEPES, 1.8 CaCl_2_, 1 MgCl_2_. pH was adjusted with TMAOH, MES replaced HEPES buffer at pH 5.0. Cells were patched in whole cell mode using Axon Multiclamp 700b amplifier; holding potential was −70 mV. Data was sampled using an Axon 1440A Digidata at 10 kHz and filtered with 100 Hz. We measured six neurons from two different preparations. Pipette solution contained (in mM): 10 NaCl, 10 KCl, 25 HEPES, 70 K-Gluconate, 10 EGTA, 1 MgCl_2_. pH was adjusted to 7.25 with TMAOH.

### Data Analysis

Data was normalized to the largest current amplitude in each measurement. For activation and steady-state desensitization curves, oocytes with a maximal current amplitude <1 μA were excluded. Using the software Igor Pro 6.02 (Wave Metrics, Inc.) and ROOT, EC_50_ and IC_50_ values and Hill-coefficients were calculated by fitting the normalized data to the Hill-equation:


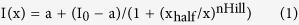


where I(x) is the current at pH x, a is the residual current, I_0_ the maximal current, x_half_ the proton concentration at which half-maximal activation occurs, and n_Hill_ the Hill-coefficient.

Steady-state desensitization curves with and without PcTx1 can, for practical reasons, only be determined with different oocytes. Therefore, we calculated the shift of steady-state desensitization curves by PcTx1 from the reduction of current by PcTx1 at one pH value. We used equation 1 fitted to the steady state desensitization curves plotted in Fig. 2, calculated the expected current at pH 7.4 for ASIC1a and 6.95 for the other channels and normalized the measured currents before PcTx1 application to these values. The measured current reduction after PcTx1 enabled us then to calculate the pH value to which the steady-state desensitization curve was shifted. The experimental error of this shift was calculated using the propagation of uncertainty.

Time constants of desensitization τ_des_ were calculated by fitting the currents at pH 4.0 to a mono-exponential function using the software Igor Pro 6.02 (Wave Metrics, Inc.). All values are reported as mean ± standard error of the mean. Significant differences between two values were determined via an unpaired students t-test and between more than two values via ANOVA using the software Prism (GraphPad software). Statistical probability is expressed as *p < 0.05, **p < 0.01, and ***p < 0.001.

### Homology Model of the concatamer ASIC1a-2a-2a

Homology models of the rASIC1a and the rASIC2a subunits were build with the software MODELLER 9.12^43^. We used the crystal structure of cASIC1 in complex with PcTx1 as a template (PDB-ID: 4FZ0)^31^. Several models were generated. The models with the lowest DOPE (Discrete Optimized Protein Energy) score were chosen for further investigation. The quality of the models were checked by a Ramachandran plot. The heterotrimer ASIC1a-2a-2a was created with the software PyMol (The PyMOL Molecular Graphics System, Version 1.7.4 Schrödinger, LLC), by aligning the single subunits onto the subunits of the cASIC1 structure in complex with PcTx1. For illustration, we kept PcTx1 in the subunit interfaces of the newly generated homology model of rASIC1a-2a-2a, based on its position in the crystal structure.

## Additional Information

**How to cite this article**: Joeres, N. *et al*. Functional and pharmacological characterization of two different ASIC1a/2a heteromers reveals their sensitivity to the spider toxin PcTx1. *Sci. Rep.* **6**, 27647; doi: 10.1038/srep27647 (2016).

## Figures and Tables

**Table 1:** Electrophysiological properties of homomeric ASIC1a, ASIC2a, oocytes co-expressing ASIC1a and ASIC2a, and 1a-2a-1a and 1a-2a-2a concatamers.

	**Activation**		**Steady state inactivation**		**τ_des_ (pH 4**.**0)**
	**EC**_**50**_	**Hill-coeff**.	**IC**_**50**_	**Hill-coeff**.	
1a	6.5 ± 0.04 (17)	2.8 ± 0.2	7.31 ± 0.004 (9)	13.9 ± 1.4	1.7 ± 0.1
2a	3.8 ± 0.1 (13)	1.0 ± 0.1	6.39 ± 0.03 (8)	2.8 ± 0.03	1.6 ± 0.1
1a/2a	5.8 ± 0.1 (12)	1.1 ± 0.1	6.82 ± 0.03 (15)	2.9 ± 0.2	0.9 ± 0.1
1a-2a-1a	5.9 ± 0.04 (14)	1.4 ± 0.1	6.91 ± 0.01 (15)	12.3 ± 1.6	1.0 ± 0.2
1a-2a-2a	5.6 ± 0.04 (13)	1.2 ± 0.1	6.85 ± 0.02 (16)	3.7 ± 0.3	0.6 ± 0.1

**Table 2:** Effect of Zn^2+^ on oocytes co-expressing ASIC1a and ASIC2a, on 1a-2a-1a and on 1a-2a-2a concatamers.

	**EC**_**50**_		**Hill-coeff**.	
	**−Zn**^**2+**^	**+Zn**^**2+**^	**−Zn**^**2+**^	**+Zn**^**2+**^
1a/2a	5.9 ± 0.1	6.1 ± 0.03	1.0 ± 0.1 (12)	1.4 ± 0.1 (10)
1a-2a-1a	5.8 ± 0.04	6.1 ± 0.02	1.4 ± 0.1 (12)	2.2 ± 0.1 (12)
1a-2a-2a	5.7 ± 0.04	5.9 ± 0.03	1.2 ± 0.1 (12)	1.1 ± 0.1 (12)
